# Accuracy and bias in the perceptions of partner’s negative emotions: the role of trait mindfulness

**DOI:** 10.1038/s41598-025-94581-2

**Published:** 2025-03-22

**Authors:** Giulia Zoppolat, Nickola Overall, Johan C. Karremans, Lara K. Kammrath, Kim Lien van der Schans, Valerie Chang, David M. Doyle, Francesca Righetti

**Affiliations:** 1 Department of Medical Psychology, Amsterdam University Medical Centers, Amsterdam, The Netherlands; 2 School of Psychology, University of Auckland, Auckland, New Zealand; 3 Behavioural Science Institute, Radboud University, Nijmegen, The Netherlands; 4 Department of Psychology, Wake Forest University, Winston-Salem, NC USA; 5 Department of Experimental and Applied Psychology, VU Amsterdam, Amsterdam, The Netherlands

**Keywords:** Trait mindfulness, Relational mindfulness, Truth and bias, Partner perceptions, Emotions, Romantic relationships, Human behaviour, Emotion

## Abstract

Theoretical and empirical work suggests that mindfulness is beneficial for close relationships. However, the ways in which mindfulness shapes important relational processes are not well understood. The current study examines the role that trait mindfulness plays in shaping people’s perceptions of their romantic partner’s emotions. In two dyadic studies (Study 1 *n* = 121 couples; Study 2 *n* = 138 couples), heterosexual couples engaged in a conversation about a problem in their relationship. Prior to the conversation, participants completed measures of their *relational* (Study 1) or *general trait* (Study 2) *mindfulness*, and then rated their own and their partner’s (negative) emotions immediately following the conflict. Multilevel Truth and Bias models were used to assess accuracy and bias in perceptions. Results revealed that men low in mindfulness overestimated their partner’s negative emotions especially when their partners’ negative emotions were higher, whereas more mindful men did not overestimate their partners’ negative emotions and were less perceptually sensitive to their partner’s higher levels of negative emotions. This pattern of results was less consistent for women. Overall, this study advances understanding of how mindfulness may enhance relationships by suggesting that mindfulness reduces hypervigilance to and overestimation of partner’s negative emotions, particularly for men.

A growing body of work has found that mindfulness—paying attention to present moment experiences in a non-judgmental manner—contributes positively to relational quality^1^. Despite growing interest in how mindfulness may enhance close relationships^2^, the processes through which mindfulness may shape relationship interactions are not yet well understood. One key way mindfulness may affect relationships is by helping partners view and interpret one another’s emotions in constructive ways, especially in challenging emotional contexts. Emotions serve an important communicative function. In particular, negative emotions indicate that a problem should be addressed, that support is needed, or that certain behavior warrants change^3,4^. Accurately perceiving negative emotions is thus crucial for managing and resolving relational issues as long as perceivers are not so vigilant that they overestimate how angry, sad, or hurt their partner is feeling^5^. In the present work we propose that, because mindfulness is an attentional process that affects how people experience social information^6^, mindfulness should play an important role in whether people are biased or accurate in perceiving their partner’s emotions.

Theoretical models of mindfulness in relationships suggest that mindfulness is associated with intrapersonal processes (e.g., ability to stay present with difficult emotions) that affect interpersonal responses, including partners’ ability to detect emotions^7^. Empirical work also supports this idea. Mindfulness involves being able to pay attention to the present moment in a non-judgmental way^8^. Accordingly, research has found that people who are more mindful are able to better regulate their emotions^9^, including the less pleasant ones, and to experience emotions without overreacting to them^10,11^. This should be particularly helpful in close interpersonal interactions. Indeed, within romantic relationships, mindfulness has been linked with greater attunement and care for partners^12^, greater empathy and perspective taking^13,14^, greater acceptance of the partner^15^, and greater self-control during conflict^10^.

Although past research supports that mindfulness may facilitate better interpersonal interactions, no previous research has examined whether people high in mindfulness are better able to detect or are less biased in perceiving emotional cues from their partner. Instead, prior studies have primarily examined emotion perceptions within stranger or acquaintance relationships, finding mixed results regarding whether mindfulness facilitates interpersonal understanding^16–21^. However, interpersonal perceptions may differ within real-life close relationships compared to perceptions of lab-based stimuli or stranger interactions. Emotions are usually more strongly felt within close relationships^22–24^. Moreover, because emotions serve a critical function and are highly consequential in close relationshps^3,4^, people are both particularly motivated to assess their partner’s emotions^25,26^ and are subject to a unique set of biases that may differently affect judgments (e.g., strong desires to maintain relationships or fears of rejection)^5,27^. Understanding whether and how mindfulness is associated with perceptions of emotions in close relationship settings is thus central to uncovering the way mindfulness is likely to shape emotion perceptions.

Fully understanding how mindfulness affects emotion perceptions also requires specifying different components of accuracy and bias^28–31^. In particular, the literature has defined three distinct but related forms of accuracy or bias: (1) directional bias (the degree to which people over- or under-estimate a partner’s negative emotion), (2) tracking accuracy (the degree to which people’s perceptions track or covary with a partner’s emotions), and (3) projection bias (projecting own emotions onto perceptions of partner’s emotions)^25,29,32^. Prior mindfulness studies have not assessed these distinct components; thus, prior inconsistent findings might be a result of assessing or muddling different types of accuracy and bias. Below we consider how mindfulness may be associated with each type of accuracy and bias. We then present two studies that test, for the first time, whether trait mindfulness is associated with accuracy *and* bias in perceptions of emotions in a highly personal and consequential domain: romantic relationship interactions.

Perceivers display “overestimation bias” or “underestimation bias” (i.e., *directional bias*) when they view their partner to be experiencing more or less negative emotions than what their partner reports. Meta-analyses have found mixed evidence as to whether perceivers generally display bias with regard to partner’s negative emotions^25,32^, suggesting that individual differences play an important role in determining levels and type of directional bias^5^. Although a partner's negative emotions are important to detect and respond to, overestimating a partner's negative emotions risks overreacting to a partner’s emotional state or escalating conflicts^33,34^. Because more mindful people tend to be better at perspective taking^14^, react with less physiological threat in conflict situations^35^, and experience feelings without overreacting to them^36^, we hypothesize that more compared to less mindful people will be less likely to overestimate their partner’s negative emotions.

Despite the potential for directional biases, perceivers generally show some degree of *tracking accuracy*^31,32^. Importantly, high levels of tracking accuracy and greater directional bias can co-occur when emotional dynamics amplify hypervigilance to a partner’s negative emotions. For example, insecure perceivers’ hyper-focus on negative emotions results in greater detection of when partners experience negative emotions (high tracking accuracy) as well as overestimation of the intensity of those emotions (high directional bias)^26,33,37^. Mindful people are more aware and observant of their present moment experiences^38,39^ and more attuned to relationship relevant information^40^, and thus may display tracking accuracy. However, because they can experience negative emotions without amplifying them^10,11^, we hypothesize that people high in mindfulness will be less likely to show a hypervigilant pattern in which tracking accuracy is accompanied by overestimating partners’ negative emotions.

*Projection bias* is measured by the association between the perceiver's own emotions and perceptions of their partner’s emotion. People strongly assume similarity when making judgements about their partner^32^. However, because more mindful people are more aware of their own emotions^41^ and can view their partner’s experiences as separate from their own^14^, we hypothesize that they should also be less inclined to project their own feelings onto their partner’s (i.e., display less projection bias) compared to less mindful people.

## Methods overview study 1 and 2

We tested whether trait mindfulness was associated with accuracy and bias in perceptions of a romantic partner’s emotions in two pre-registered dyadic studies. In Study 1 (*n* = 121 couples in the Netherlands) we tested the links between *relational mindfulness*, which reflects trait mindfulness in interactions with relationship partners, and perceptions of partners’ anger repeatedly assessed during couples’ discussions of a conflict of interests between partners. In Study 2 (*n* = 138 couples in New Zealand) we examined the links between *trait mindfulness*, using the widely used Five Facet Mindfulness Questionnaire (FFMQ)^38^ to capture people’s broader mindfulness tendencies, and perceptions of partners’ negative emotions (e.g., anger, hurt) rated once immediately following couples’ conflict discussions. Testing the effects of mindfulness on accuracy and bias in two couple interaction studies drawn from different populations, engaging in different conflictual conversations (divergence of interests; serious relationship issue), and examining two different types of trait mindfulness (relational and general), enables assessment of whether the effects are robust and replicate across different populations, couple discussions, and mindfulness measures.

In both studies, we also explored (Study 1) and conducted pre-registered tests (Study 2) of gender as a possible moderator because research has shown gender can sometimes shape partner perceptions^25,26^, and a recent meta-analysis indicates that men may be more likely to show negative (i.e., overestimation) biases^32^. Further, because people’s general relationship sentiments and attachment insecurities can shape partner perceptions^5,42^ and are related and overlap in their outcomes with mindfulness^43,44^, we performed auxiliary analyses controlling for these variables to ensure that the effects in both studies were not simply driven by how satisfied and secure people felt in their relationship. Both studies were approved by the Ethical Review Board of the host institutions (Vrije Universiteit Amsterdam and University of Auckland) in accordance with institutional and national regulations and all participants provided informed consent. Pre-registrations and code are available and can be accessed at the project’s OSF page: https://osf.io/z25vw/?view_only=63e1ff81850445f99f9696a1501f2d23

### Participants and procedure study 1

Data were collected from 130 couples living in the Netherlands. One participant and six couples were excluded for not following directions, and one same-gender couple was excluded given examination of the data revealed gender differences (see below for results of distinguishability analyses). The final sample size was 121 couples. Participants were on average 23.38 years old (*SD* = 3.66). Relationships were on average 2.8 years in length *(SD* = 29 months, range 4 months–17 years) and 35% couples were cohabitating.

Couples attended an in-person research session. After independently completing baseline questionnaires, including demographic information and an assessment of their relational mindfulness, relationship satisfaction, and attachment security, couples engaged in a 7-min video-taped conversation in a private room in which they discussed a divergence of interest that they were currently experiencing in their relationship. Couples were given common examples of divergence of interests prior to the conversation (e.g., whose family to visit for the holidays, how to spend the weekend, whether to move to a different country for a job opportunity). Further information about the conversation procedure can be found in the Supplemental Material (SM). Immediately following the conversation, participants were separated and viewed the video of their conversation, rating their own and perceptions of their partner’s emotions in 30-s intervals (for a total of 14 repeated assessments per participant).

### Measures study 1

See the SM for full items and scale information. See Table 1 for descriptive statistics.

**Table 1:** Means, standard deviations, and correlations in Study 1 and 2.

	*M*	*SD*	1	2	3	4	5	6
Study 1								
1. Relational mindfulness	5.91	0.69	1					
2. Relationship satisfaction	5.98	0.83	0.26**	1				
3. Attachment anxiety	2.53	0.92	− 0.18**	− 0.13**	1			
4. Attachment avoidance	3.36	0.90	− 0.07**	− 0.05**	0.13**	1		
5. Perceiver anger	1.69	1.08	− 0.13**	− 0.17**	0.19**	0.01	1	
6. Partner anger	1.69	1.08	− 0.08**	− 0.08**	0.07**	− 0.03	0.24**	1
7. Perceived partner anger	1.68	1.11	− 0.17**	− 0.16**	0.12**	0.02	0.61**	0.30**
Study 2								
1. Trait mindfulness	3.23	0.46	1					
2. Relationship satisfaction	6.07	0.82	0.08	1				
3. Attachment anxiety	3.05	1.10	− 0.28**	− 0.22**	1			
4. Attachment avoidance	2.87	0.98	− 0.05	− 0.38**	0.19**	1		
5. Perceiver negative emotions	1.63	1.15	− 0.06	− 0.39**	0.21**	0.10	1	
6. Partner negative emotions	1.63	1.15	− 0.05	− 0.33**	0.16**	0.15*	0.53**	1
7. Perceptions of partner negative emotions	1.78	1.23	− 0.15*	− 0.36**	0.21**	0.17**	0.71**	0.60**

All variables were assessed on a scale of 1–7, except Mindfulness was assessed on a scale 1–5 in Study 2. Correlations represent zero-order correlations. Correlations with emotion variables in Study 1 were calculated across all measurement time points. *p < 0.05 **p < 0.01.

*Relational mindfulness* was measured at intake using a newly developed measure to capture mindful presence in relationships (13-items, e.g. “I think of other things when my partner is speaking to me,” “I find myself listening to my partner with one ear, while doing something else at the same time,” reverse scored; α = 0.90) rated on a 7-point Likert Scale (1 = *always*, to 7 = *never*). The original scale comprised 15 items, but two items were dropped according to pre-registered criteria based on factor loading of less than 0.40 (see SM).

Established scales assessed *relationship satisfaction* (four items, e.g., “I feel satisfied with our relationship”; α = 0.82)^45^, *attachment anxiety* (five items, e.g., “I often worry that my partner(s) don’t really love me”; α = 0.61), and *attachment avoidance* (eight items, e.g., "I'm not very comfortable having to depend on other people”; α = 0.73)^46^, all rated on 7-point scales (1 = *totally disagree* to 7 = *completely agree*).

During the review of their conversation participants rated their own anger (“In that moment, how angry did you feel?”) as well as *perception of their partner’s* anger (“In that moment, how angry was your partner?) measured on a 7-point scale (1 = *Not at all* to 7 = *Extremely*). Comparable items assessed stress and happiness (see SM). We focus on anger in the main manuscript and briefly report the additional pre-registered tests for stress and happiness in the SM. We chose this reporting approach to focus on negative emotions directed toward the perceiver as assessed and pre-registered in Study 2 because these more threatening emotions underpin the patterns of bias and accuracy covered in the Introduction.

### Analytical strategy study 1

We applied multilevel Truth and Bias Models to assess bias and accuracy for repeated measures dyadic data using the MIXED procedure in SPSS 28^5,29^ (see OSF for syntax). This approach tests whether participants exhibit *directional bias* (perceive their partner’s emotions to be lower or higher than partners’ reported negative emotions), *tracking accuracy* (detect variations in their partner’s emotion across each 30-s section of the discussion), and *projection bias* (perceive their partner’s emotions to be similar to their own). Our models also tested whether relational mindfulness predicted levels of directional bias, tracking accuracy, and projection bias. The Truth and Bias Models involved regressing perceptions of partners’ emotion on (a) partners’ actual emotion, (b) perceivers’ own emotion, (c) relational mindfulness, (d) the interaction term between relational mindfulness and the partners’ actual emotions, and (e) the interaction term between relational mindfulness and the perceivers’ own emotions. All emotion scores were centered around the partners’ actual emotion^29,47^. This centering approach means that the intercept represents perceivers’ *directional bias*; a negative intercept indicates underestimation of partners’ emotions on average, a positive intercept indicates overestimation of partners’ emotions on average, and a non-significant intercept indicates no directional bias^29,47^. The coefficient of the partners’ actual emotion represents perceivers’ *tracking accuracy* across the discussion; a higher positive coefficient indicates more accuracy in tracking the partners’ emotional variations throughout the conversation. The coefficient of the perceivers’ own emotion represents *projection bias*; a higher coefficient indicates a greater tendency for perceivers who experienced more negative emotions to assume their partner also was experiencing more negative emotions. The main effect of perceivers’ relational mindfulness (grand-mean centered) is *the effect of relational mindfulness on directional bias*, the interaction term of relational mindfulness and the partners’ actual emotion is the *effect of relational mindfulness on tracking accuracy*, and the interaction term of mindfulness and perceivers’ own emotion is the *effect of relational mindfulness on projection bias*.

Distinguishability analyses^48^ revealed that the relations between parameters differed by gender due to unequal means *X*^2^ (3) = 27.67, *p* < 0.001 and variances *X*^2^ (3) = 10.46, *p* = 0.015. Thus, we then tested models with gender (coded as women = 1, men = − 1) as a moderator of all model coefficients described above. When a significant effect of gender was present, no-intercept multilevel models were conducted^49^ which simultaneously estimates separate effects for men and women while accounting for the dependence across partners. Finally, we conducted additional analyses controlling for relationship satisfaction or attachment anxiety and avoidance (grand-mean centered) to test whether the effects of relational mindfulness occurred above and beyond people’s general feelings about their relationship and their attachment insecurities.

### Results study 1

The results examining perceptions of partner’s anger are presented in Table 2. Mindfulness was significantly negatively associated with directional bias. Calculating directional bias at high (+ 1 SD) vs. low (-1 SD) levels of mindfulness revealed that perceivers low in mindfulness significantly overestimated their partner’s anger (*b* = 0.13, *SE* = 0.06, *t* = 2.04, *p* = 0.044), whereas perceivers high in mindfulness significantly underestimated their partner’s anger (*b* = − 0.18, *SE* = 0.07, *t* = − 2.76, *p* = 0.007). Mindfulness was not significantly associated with tracking accuracy, but was significantly negatively associated with projection bias. Calculating projection bias at high (+ 1 SD) vs. low (-1SD) mindfulness revealed that projection bias was stronger for people lower (*b* = 0.41, *SE* = 0.04, *t* = 10.712, *p* < 0.001) compared to higher (*b* = 0.28, *SE* = 0.04, *t* = 7.089, *p* < 0.001) in mindfulness.

**Table 2:** Directional bias, tracking accuracy, and projection in the perception of a partner’s anger (study 1) and negative emotions (study 2) during conflict conversations.

Bias and accuracy of perception partner’s emotions	*b*	95% CI	*t*	*p*
Study 1				
Directional bias	− 0.02	[− 0.12, 0.07]	− 0.473	0.638
Tracking accuracy	0.08	[0.04, 0.12]	3.834	< 0.001
Projection	0.35	[0.30, 0.39]	13.849	< 0.001
Effects of mindfulness				
Directional bias	− 0.23	[− 0.36, − 0.10]	− 3.454	< 0.001
Tracking accuracy	− 0.06	[− 0.13, 0.02]	− 1.491	0.138
Projection	− 0.10	[− 0.18, − 0.01]	− 2.169	0.032
Study 2				
Directional bias	0.11	[0.03, 0.19]	2.631	0.010
Tracking accuracy	0.31	[0.21, 0.41]	6.198	< 0.001
Projection	0.59	[0.49, 0.68]	12.054	< 0.001
Effects of mindfulness				
Directional bias	− 0.25	[− 0.43, − 0.06]	− 2.617	0.009
Tracking accuracy	− 0.40	[− 0.61, − 0.18]	− 3.635	< 0.001
Projection	− 0.06	[− 0.24, 0.13]	− 0.603	0.547

**Fig. 1:**
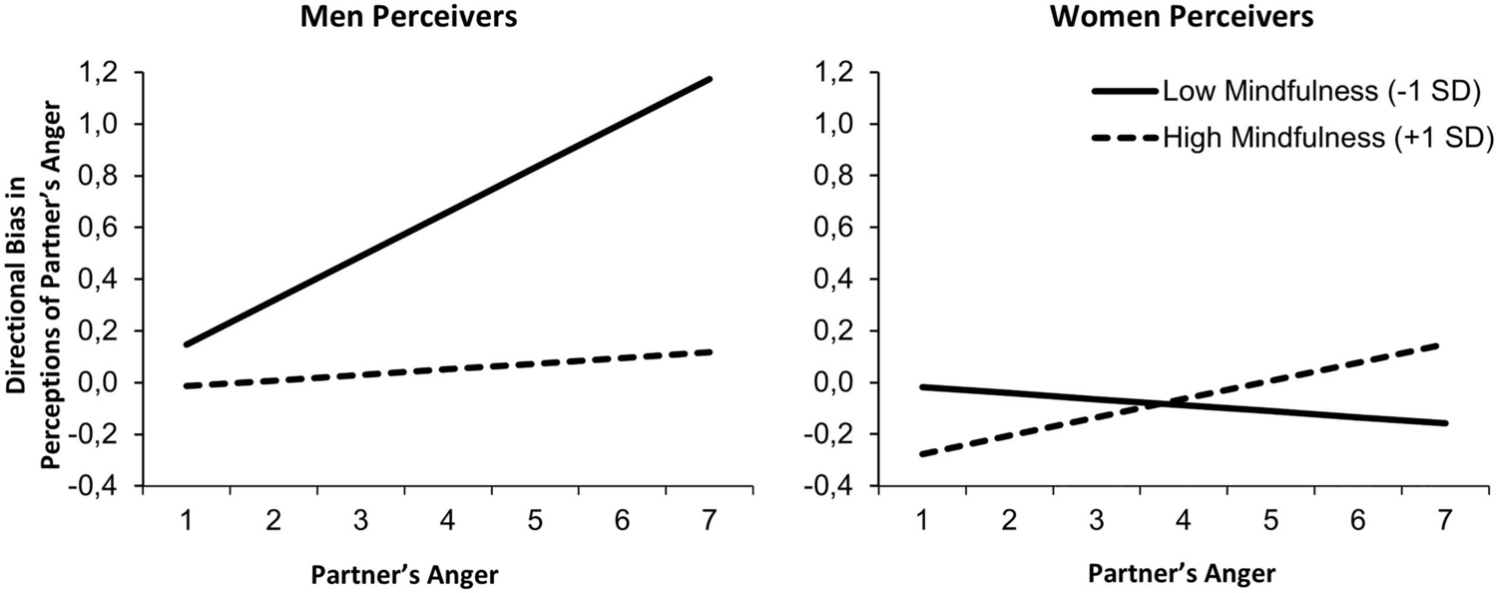
The effects of mindfulness on perceptions of partner’s anger (y axis centered to represent directional bias) across levels of partner’s anger (x axis representing tracking accuracy) for men (left pane) and women (right pane) perceivers high (+ 1SD) versus low (− 1SD) in mindfulness.

We then added the main and all interaction effects of gender into the model to test whether gender moderated the effects of mindfulness. The first column of Table 3 shows the tests of whether gender moderated each effect. Levels of directional bias and projection bias significantly differed across men and women perceivers (see top left of Table 3). Additionally, the effects of mindfulness on tracking accuracy significantly differed across men and women (see gender moderation of effects of mindfulness on tracking accuracy in Table 3). Figure 1 illustrates the gender differences of mindfulness on directional bias and tracking accuracy. Focusing first on men, men low in mindfulness significantly overestimated their partner’s negative emotions on average (scores on average above zero; *b* = 0.27, *SE* = 0.10, *t* = 2.56, *p* = 0.012) whereas men high in mindfulness did not (scores on average did not differ from zero; *b* = 0.001, *SE* = 0.10, *t* = 0.01, *p* = 0.989). Additionally, men low in mindfulness showed significant tracking accuracy (i.e., perceiving partner’s anger to be higher as partner’s anger was higher, *slope* = 0.17, *SE* = 0.04, *t* = 4.826, *p* < 0.001), whereas men high in mindfulness did not (*slope* = 0.02, *SE* = 0.05, *t* = 0.405, *p* = 0.686). Figure 1 demonstrates the implications for understanding the links between mindfulness and directional bias. Differences across levels of mindfulness became greater the more partners were experiencing anger such that men low (but not high) in mindfulness were increasingly likely to overestimate their partner’s anger when their partner was experiencing higher anger.

**Table 3:** Accuracy and bias of anger (Study 1) and negative emotions (Study 2) across men and women perceivers.

Perception of partner’s negative emotions	Gender moderation				Men perceivers				Women perceivers			
	*b*	t	95% CI	*p*	*b*	t	95% CI	*p*	*b*	t	95% CI	*p*
Study 1												
Directional bias	− 0.13	− 2.900	[− 0.22, − 0.04]	0.004	0.13	1.846	[− 0.01, 0.28]	0.068	− 0.13	− 2.136	[− 0.25, − 0.01]	0.035
Tracking accuracy	− 0.04	− 1.490	[− 0.08, 0.01]	0.140	0.10	3.129	[0.04, 0.16]	0.068	0.02	0.660	[− 0.05, 0.10]	0.513
Projection	− 0.08	− 2.433	[− 0.15, − 0.02]	0.017	0.50	8.781	[0.38, 0.61]	< 0.001	0.33	9.702	[0.26, 0.40]	< 0.001
Effects of mindfulness												
Directional bias	0.03	0.367	[− 0.11, 0.16]	0.714	− 0.19	− 1.724	[− 0.41, 0.03]	0.088	− 0.14	− 1.676	[− 0.31, 0.03]	0.097
Tracking accuracy	0.09	2.155	[0.01, 0.17]	0.034	− 0.11	− 2.240	[− 0.21, − 0.01]	0.028	0.07	1.026	[− 0.07, 0.20]	0.310
Projection	− 0.09	− 1.813	[− 0.19, 0.01]	0.072	0.04	0.558	[− 0.11, 0.20]	0.579	− 0.13	− 2.232	[− 0.25, − 0.02]	0.028
Study 2												
Directional bias	0.14	2.776	[0.04, 0.25]	0.006	0.30	4.055	[0.15, 0.44]	< 0.001	0.01	0.171	[− 0.11, 0.13]	0.864
Tracking accuracy	0.02	0.339	[− 0.08, 0.11]	0.735	0.31	4.504	[0.17, 0.45]	< 0.001	0.28	3.943	[0.14, 0.42]	0.864
Projection	0.06	1.261	[− 0.04, 0.16]	0.209	0.70	8.161	[0.53, 0.89]	< 0.001	0.58	10.267	[0.47, 0.69]	< 0.001
Effects of mindfulness												
Directional bias	− 0.03	− 0.236	[− 0.23, 0.18]	0.813	− 0.23	− 1.409	[− 0.56, 0.09]	0.161	− 0.18	− 1.485	[− 0.43, 0.06]	0.140
Tracking accuracy	− 0.25	− 2.220	[− 0.47, − 0.03]	0.027	− 0.74	− 4.489	[− 1.07, − 0.41]	< 0.001	− 0.24	− 1.540	[− 0.54, 0.07]	0.125
Projection	0.29	2.570	[0.07, 0.52]	0.011	0.41	2.032	[0.01, 0.80]	0.044	− 0.17	− 1.640	[− 0.39, 0.04]	0.104

The first column of this table shows the coefficients testing whether gender moderated the effects, and the second and third columns illustrate the effects for men and women perceivers.

Gender differences in levels of bias and the effects of mindfulness on tracking accuracy revealed a different pattern for women. Women low in mindfulness did not show directional bias (scores on average did not differ from zero; *b* = − 0.03, *SE* = 0.09, *t* = − 0.37, *p* = 0.715) or tracking accuracy (non-significant flat slope across partners’ levels of anger; *b* = − 0.03, *SE* = 0.07, *t* = − 0.44, *p* = 0.728). By contrast, women high in mindfulness tended to underestimate their partner’s negative emotions (scores on average below zero; *b* = − 0.23, *SE* = 0.07, *t* = − 3.06, *p* = 0.003) but did not show significant tracking accuracy (*b* = 0.07, *SE* = 0.05, *t* = 1.43, *p* = 0.162).

#### Additional analyses

The pattern of results of mindfulness on accuracy and bias described above remained unaltered when controlling for global relationship quality or attachment anxiety and avoidance. See SM for full results. Additionally, we ran the same models with perceptions of partner’s stress. Mindfulness was again significantly negatively associated with directional bias resulting in perceivers low in mindfulness significantly overestimated their partner’s stress, whereas perceivers high in mindfulness did not show directional bias. This effect did not differ across gender. No effects of mindfulness on projection bias or tracking accuracy emerged. Finally, no effects of mindfulness were found for perceptions of happiness. See SM for full results.

### Methods study 2

The results for Study 1 provided initial evidence that mindfulness was associated with less bias in perceptions of partner’s negative emotions, particularly for men. We conducted a second study with couples to test the replicability of the results in Study 1 in another conflictual setting and using a general measure of trait mindfulness. We pre-registered (1) the hypothesis that mindfulness would be associated with less biased perceptions of a partner's negative emotions and (2) replication tests of the gender differences found in Study 1.

### Participants and procedure study 2

Data were collected from 143 couples living in New Zealand. As pre-registered, given one aim was to examine gender differences, same-gender couples (*n* = 5) were excluded from analyses. The final sample size was 138 couples. Participants were 24.8 years old on average (*SD* = 7.5) and in a serious relationship with their partner (51.5% reported living together or being married) for an average of 3.6 years (*SD* = 5.10 years).

Couples attended a lab-based session where they individually completed measures assessing demographic information, trait mindfulness, relationship satisfaction, and attachment security. After a warm-up discussion about non-conflictual events in the past week, couples had a video-recorded 7-min discussion about a serious ongoing issue in their relationships, chosen by one of the couple members (whose topic was chosen prior to the research session and counterbalanced across gender). Participants were asked to engage in the conversation as they would normally. See SM for details about the conversation procedure. Couples were then separated, and participants independently rated their own and their partner’s emotions during the conflict conversation.

### Measures study 2

See SM for full measures and Table 1 for descriptive statistics.

*Mindfulness* was measured using the commonly used FFMQ (Five Facet Mindfulness Questionnaire)^38^ measure of trait mindfulness (39-items; e.g. “I perceive my feelings and emotions without having to react to them”) rated on a 5-point Likert Scale (1 = *Never or very rarely true*, to 5 = *Very often or always true*) (α = 0.88). Because we were interested in participants’ overall average levels of mindfulness, mindfulness was calculated as the average score across all items following previous studies^50,51^. However, given an ongoing discussion regarding the validity of the *observing* facet of the FFMQ^52,53^, we conducted and present in the SM auxiliary analyses excluding this facet from the full mindfulness score. The general pattern of results and conclusion were the same.

Participants completed the same measures of *relationship satisfaction* (α = 0.82), *attachment anxiety* (α = 0.81) and *attachment avoidance* (α = 0.82) as in Study 1. Following the conversation, each participant rated their own (“I felt angry/annoyed at my partner”, “I felt hurt/rejected by my partner”, “I felt disappointed by my partner”, “I felt frustrated with my partner”) and their perception of their partner’s negative emotions (“My partner felt angry/annoyed at me”, “My partner felt hurt/rejected by me”, “My partner felt disappointed in me”, “My partner felt frustrated with me”) on 7-point scales from 1 = *Not at all* to 7 = *Very much.* As pre-registered, the four negative emotions were combined to index *own negative emotions* (α = 0.93) and *perception of partner negative emotion* (α = 0.91).

### Analytical strategy study 2

Multilevel Truth and Bias models were conducted as in Study 1. Unlike Study 1, emotions were only assessed once. Thus, the meaning of directional bias and projection bias remain the same, but tracking accuracy refers to whether perceivers detect greater negative emotions in partners who report more negative emotions compared to other partners in the sample (rather than accurately tracking varying levels of partners’ negative emotions across time as in Study 1). As in Study 1, distinguishability analyses^48^ revealed unequal means *X*^2^ (3) = 27.67, *p* = 0.034 and variances *X*^2^ (3) = 27.67, *p* = 0.009 between men and women, and thus (as pre-registered) we tested whether the effects differed by gender (coded as men = 1, women = -1).

### Results study 2

The results are presented in Table 2. Mindfulness was significantly negatively associated with directional bias. Perceivers low in mindfulness (− 1 SD) significantly overestimated their partner’s negative emotions (*b* = 0.22, *SE* = 0.06, *t* = 3.73, *p* =  < 0.001), whereas perceivers high in mindfulness (+ 1 SD) did not (*b* = − 0.01, *SE* = 0.06, *t* = − 1.22, *p* = 0.903). Additionally, mindfulness was not significantly associated with projection bias. Mindfulness also was significantly associated with tracking accuracy, but because these effects were moderated by gender (tested next), we present the overall effects across men and women in the SM and detail the effects by gender below.

**Fig. 2:**
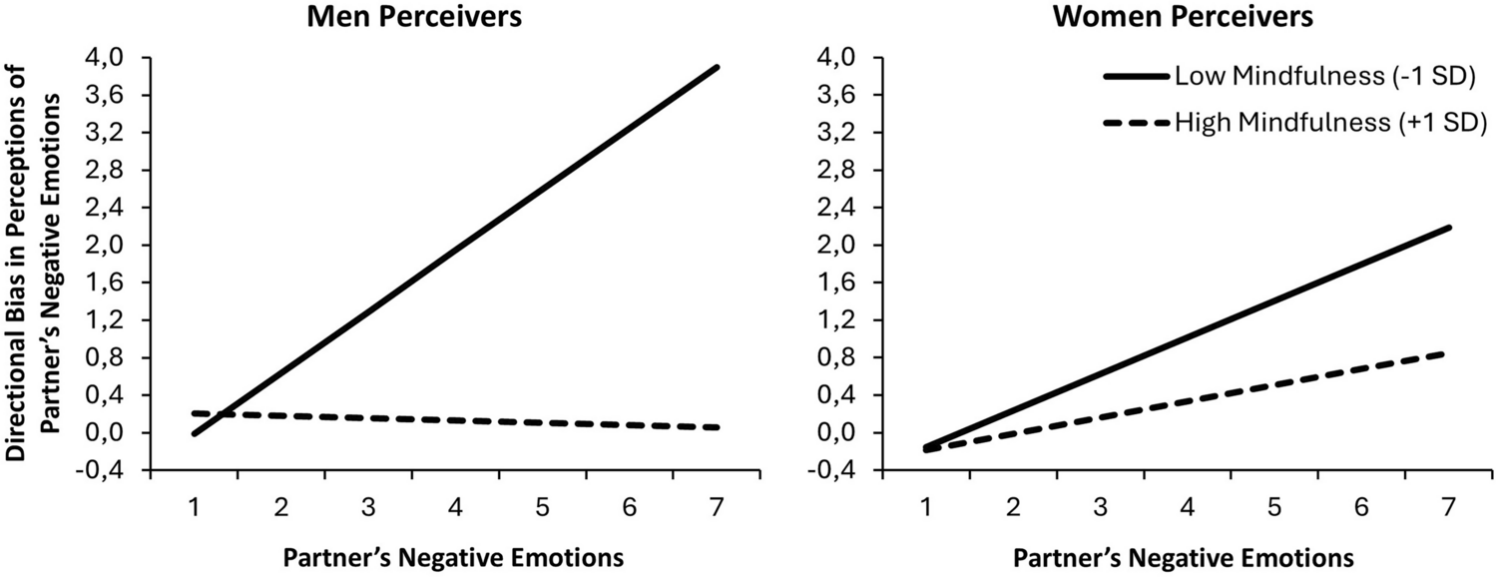
The effects of mindfulness on men’s (left panel) and women’s (right panel) perceptions of partner’s negative emotions across levels of partner’s negative emotions.

Models adding the main and all interaction effects of gender are shown in the bottom section of Table 3. Similarly to Study 1, significant gender differences emerged in levels of directional bias and the effect of mindfulness on tracking accuracy (see gender moderation effects bottom left of Table 3). Figure 2 plots the gender differences of mindfulness on directional bias and tracking accuracy, which illustrate that the effects of mindfulness on bias and tracking accuracy mostly emerged for men. Focusing first on men, replicating Study 1, men low (− 1 SD) in mindfulness significantly overestimated their partner’s negative emotions on average (scores on average above zero; *b* = 0.40, *SE* = 0.10, *t* = 3.86, *p* < 0.001) whereas men high (+ 1 SD) in mindfulness did not (scores on average did not differ from zero; *b* = 0.19, *SE* = 0.11, *t* = 1.78, *p* = 0.077). And, again similar to Study 1, men low in mindfulness showed significant tracking accuracy (*slope* = 0.65, *SE* = 0.10, *t* = 6.433, *p* < 0.001), whereas men high in mindfulness did not (*slope* = -0.03, *SE* = 0.10, *t* = − 0.237, *p* = 0.813). Thus, men low (but not high) in mindfulness were increasingly likely to overestimate their partner’s negative emotions as their partners experienced higher levels of negative emotions.

As shown in the right panel of Fig. 2, the pattern for women was less pronounced. Neither women low (*b* = 0.90, *SE* = 0.08, *t* = 1.21, *p* = 0.227) or high (*b* = -0.07, *SE* = 0.09, *t* = − 0.86, *p* = 0.392) in mindfulness exhibited significant directional bias on average. However, women low (*slope* = 0.39, *SE* = 0.08, *t* = 4.91, *p* < 0.001) but not high (*slope* = 0.17, *SE* = 0.12, *t* = 1.48, *p* = 0.144) in mindfulness demonstrated tracking accuracy. As shown in Fig. 2, women low in mindfulness were increasingly likely to overestimate their partners’ negative emotions as partners increasingly experienced higher levels of negative emotions, but differences across low versus high levels of mindfulness were smaller than for men.

Finally, the effect of mindfulness on projection bias also differed across men and women (see gender moderation effects bottom left of Table 3). As shown by the specific effects for men and women (Table 3 middle and right section, bottom row), mindfulness was significantly associated with projection bias for men but not for women. However, the pattern differed from Study 1; levels of projection bias tended to be stronger for men high (+ 1 SD; *slope* = 0.88, *SE* = 0.14, *t* = 6.271, *p* < 0.001) compared to low (− 1 SD; *slope* = 0.51, *SE* = 0.10, *t* = 4.942, *p* < 0.001) in mindfulness suggesting that, unlike in Study 1, men in Study 2 tended to be more likely to assume their partner feels the same they do.

#### Additional analyses

As in Study 1, the effects of mindfulness on accuracy and bias described above remained unaltered when rerunning the analyses controlling for relationship satisfaction or attachment insecurity. See SM for full results.

## General discussion

The current studies examine, for the first time, whether relational and trait mindfulness were associated with accuracy and bias in perceptions of partners’ negative emotion during couples’ interactions. A consistent pattern replicated across studies. Men low in mindfulness were more likely to overestimate their partners’ negative emotions—perceiving their partner’s negative emotions to be higher than they were—and display higher levels of tracking accuracy—perceiving their partners’ negative emotions to be higher when partners were reporting higher negative emotions. This pattern of directional bias and accuracy is associated with threat and insecurity in relationships^26,33^ and reflects a greater sensitivity in detecting the presence of partner’s more intense negative emotions but also overestimating the intensity of those negative emotions. More mindful men did not show this mix of tracking accuracy and bias suggesting that greater relational or trait mindfulness helps limit men’s reactance to partners’ negative emotional states (reducing tracking accuracy) and avoids perceiving partner’s negative emotions as more intense than they are (reducing overestimation bias).

The pattern of directional bias and tracking accuracy was less consistent for women. In Study 1, women high in relational mindfulness underestimated their partner’s anger. In Study 2, however, the pattern of effects were the same as men, although the differences between high versus low trait mindfulness were less pronounced. Recent meta-analytic evidence suggests that men generally show more negative biases in relationships than women^32^. These gender differences may be more likely to occur for negative emotions due to socialization processes that create differences in comfort with and regulation of emotions and expectations around managing emotive interactions^54,55^. Accordingly, men may be more vulnerable to discomfort and reactivity to their partner’s negative emotions within conflictual interactions, and therefore mindfulness may be particularly helpful by reducing men’s greater sensitivity to partner’s negative emotions. This explanation also is consistent with prior findings that mindfulness is associated with greater constructive problem-solving strategies for men but not for women^56^. This gender difference may be because mindfulness promotes greater perspective taking, and perspective taking may make a bigger difference for men who tend to report lower levels of empathy^57^ and are generally expected to perform less emotional work in relationships^55^ than women. Given previous research has shown that cultivating mindfulness (e.g., through contemplative practices) can lead to changes in dispositional mindfulness^58^, future research could examine whether contemplative practices may have more benefits in reducing men's biased perceptions. Additionally, future studies could measure and identify relevant behavioral processes that may relate to mindfulness and emotion perception, including gender differences, such as active listening, communication, or expression of emotions.

The effects of mindfulness on projection bias were inconsistent across studies. Higher relational mindfulness was associated with lower projection bias by both men and women in Study 1, suggesting that mindfulness may reduce the degree to which people assume their partner experiences the same emotions they do. However, in Study 2, men’s (but not women’s) higher trait mindfulness was associated with greater projection bias. These differences may relate to the different measures of mindfulness and emotions. Perhaps people who are specifically mindful about their relationship (Study 1) are equally or more reflective of their partners’ specific emotions (like anger) as their own, reducing projection bias. By contrast, trait mindfulness not specific to relationships (Study 2) may capture a greater relative mindful focus on people’s own emotions, which may encourage or even accentuate typical projection tendencies especially of more general emotional experiences (across a range of negative emotions). Notably, perceivers showed strong projection bias regardless of levels of mindfulness. This is consistent with perceivers generally showing high levels of projection bias^32^,which may not be easily or strongly modified by individual differences such as mindfulness. Future research is needed to better understand the potential links between mindfulness and projection bias.

The present studies build upon and advance the literature on mindfulness and emotion perception in relationships in several ways. First, theoretical accounts of mindfulness in relationships have proposed that mindfulness should influence interpersonal experiences due to the effects that mindfulness has on intrapersonal processes^7^. The present work suggests that a way in which mindfulness may do so is by shaping perception of negative emotions, and in particular reducing sensitivity to and overestimation of partners’ negative emotions.

Second, the present work also provides some nuance to the positivity hypothesis of mindfulness^59^. In general, higher mindfulness did not predict positive bias, such as perceiving partners’ negative emotions to be lower than they were (with one exception for women’s perceptions of anger in Study 1). Additional analyses in Study 1 also indicated that people high in mindfulness did not overestimate happiness. Thus, although our results indicate that mindfulness may help attenuate stressful situations by not exaggerating the negative, this does not generally appear to result in mindful people showing rose-colored glasses. This is important because perceptions can significantly shape how people behave within relationship interactions^5,25,31^ and unbiased perceptions of partner’s negative emotions are likely essential for promoting appropriate and constructive relational responses^5^.

Third, the present work brings clarity to conflicting prior evidence as to whether mindfulness improves interpersonal understanding. It is possible that mixed findings in the literature are because prior research has used varying indices of accuracy that have not distinguished between tracking accuracy, directional bias, and projection bias^18,19^. Failing to simultaneously measure each bias masks important information on the direction of bias—whether mindful people over or underestimate negative emotions—and how bias emerges as part of other perceptual processes, such as tracking accuracy or projection bias. Testing the key components of bias and accuracy provides important insight into the aspects of emotion perceptions likely affected by mindfulness. In the current studies, compared to perceivers low in mindfulness, men (and women in Study 2) higher in relational and trait mindfulness were less likely to overestimate their partners’ negative emotions, in part because they were less perceptually reactive when partners were experiencing higher levels of negative emotions.

Future research should also address the limitations of the current work. Although relational and trait mindfulness showed the same replicated pattern with regard to men’s directional bias and tracking accuracy, relational and trait mindfulness were not measured within the same study, preventing tests of their unique effects. Relational mindfulness mainly captures mindful presence in relationships, suggesting that the Study 1 effects may be due to the attentional component of mindfulness within the specific relational context. The FFMQ in Study 2 captures five facets of mindfulness, suggesting that the replicated effects for men’s accuracy and bias in Study 2 are likely generalizable to a broader conceptualization of mindfulness. However, the use of different scales could contribute to some of the differences across studies, such as varying patterns of projection bias. Understanding the distinctions and relevance of different types of mindfulness and their contribution to relationship perceptions is an important future research direction.

Our studies also included relatively happy couples and couples’ conversations did not involve especially high levels of negative emotions (as is typical in lab-based studies)^5,37^. According to the stress buffering hypothesis of mindfulness, mindfulness should be especially beneficial in stressful situations^60^. Results from the present work support this idea as differences in the effects of mindfulness on perceptions became increasingly stronger as partners experienced higher levels of negative emotions, with more mindful people less likely to overestimate their partners’ negative emotions. However, assessing whether mindfulness reduces more biased perceptions of a partner’s emotions in more distressed couples and emotionally intense interactions would be a valuable future direction. Further, the different effects of mindfulness for men versus women were generally consistent for perceiving specific negative emotions (anger) and a composite of negative emotions (e.g., anger, frustration) directed toward the self. However, future work could assess whether the effects of mindfulness and any gender differences vary according to partners’ emotions about themselves (e.g., pride, shame) which may be less threatening.

Finally, these limitations are balanced by important strengths, including examining real-life interactions to provide rich, ecologically-valid data that adds to very few mindfulness studies examining actual interactions between romantic partners^35,61^. Moreover, we found a replicated pattern across studies assessing relational mindfulness and general trait mindfulness, responding to the call by researchers^62,63^ to consider contextual factors in which mindfulness processes are taking place while providing evidence for generalizability. Overall, this novel investigation suggests that mindfulness helps people, particularly men, experience their partner’s higher negative emotions more as they actually are, without exaggerating them and without hypervigilance to negative emotional cues. These findings provide new insight into the role that mindfulness can play in emotive relational contexts.

## Supplementary Information

Supplementary Information.

## Data Availability

The anonymized data to reproduce the analyses has been uploaded to a secure data repository (https://dataverse.nl/privateurl.xhtml?token=c71c580f-fafe-4855-80ff-0317c35cf5bf). The data can be accessed during the review process. Given the sensitive nature of dyadic data and the data privacy regulations of the host institutions, upon publication of the manuscript, the data will be available through the secure data repository by emailing the first author. Pre-registrations and code are available and can be accessed at the project’s OSF page: https://osf.io/z25vw/?view_only=63e1ff81850445f99f9696a1501f2d23.
