# Supplementary material for: Accuracy and bias in the perceptions of partner’s negative emotions: the role of trait mindfulness
Source: Sci Rep. 2025 Mar 22;15:9977. doi: 10.1038/s41598-025-94581-2 (PMC11929810; doi:10.1038/s41598-025-94581-2)
Supplement: Supplementary file 1 — Supplementary Information. [file 41598_2025_94581_MOESM1_ESM.pdf]

Supplemental Material

Accuracy and Bias in the Perceptions of Partner's Negative Emotions: The Role of Trait Mindfulness

Giulia Zoppolat, Nickola Overall, Johan C. Karremans, Lara K. Kammrath, Kim Lien van der Schans, Valerie Chang, David M. Doyle, Francesca Righetti

Table of Content

Study 1

1. Relational Mindfulness scale
2. Stress and Happiness Items
3. Stress and Happiness Accuracy and Bias Results
4. Analyses controlling for relationship satisfaction and attachment styles

Study 2

5. Accuracy and bias across gender results
6. Analyses controlling for relationship satisfaction and attachment styles
7. Results from supplemental analyses conducted using the FFMQ without the *observing* subscale

Study 1 and 2

8. Relational measures Study 1 and 2
9. Additional methodological information on laboratory conversation

Study 1

1. Relational Mindfulness Scale

The full scale consisted of fifteen items. Correlation matrix revealed correlations above .30 but below .80, and the determinant of the correlation matrix was .001, suggesting multicollinearity was likely not an issue. Factor analysis was conducted according to recommendations by Yong & Pearce, 2013. Items 3 and 14 (see scale below) showed loading of less than .40. Following pre-registered criteria, these items were dropped. Results also revealed two underlying factors. However, as pre-registered, decisions about the scale would include an assessment by the research team based on theoretical considerations and face validity, and a determination was made to use all remaining items as a single scale.

1) I think of other things when my partner is speaking to me.
2) I tend not to notice things that are going on with my partner until my partner tells me about them.
3) ~~I rush through activities with my partner without being really attentive to him or her.~~
4) I find myself listening to my partner with one ear, while doing something else at the same time.
5) I have conversations on "automatic pilot" with my partner and then wonder what we talked about later.
6) I find myself preoccupied with the past or future when I'm with my partner.
7) I tend to forget that my partner is present.
8) I tend not to notice when my partner changes something about his/her physical appearance.
9) My partner has to repeat himself or herself to me, because I wasn't really paying attention the first time.
10) I tend not to really pay attention when my partner tells me about a problem.
11) I tend not to notice changes in my partner's health or well-being right away.
12) It takes me awhile to notice when my partner is upset or frustrated about something.
13) I tend not to notice when my partner has done something nice for me.
14) ~~I tend not to notice when my partner has achieved something.~~
15) I tend to say, "yes," and "uh huh," to my partner without really listening to him or her.

2. Stress and Happiness Items and Descriptive Statistics in Study 1

In addition to anger (as reported in the Manuscript), participants also rated how stressed ("In that moment, how stressed did you feel?"; *M* = 1.88, *SD* = 1.21) and happy they felt ("In that moment, how happy did you feel?"), as well as perceptions of their partner's anger ("In that moment, how stressed was your partner") and happiness ("In that moment, how happy was your partner?"). Below we present all means, standard deviations, and correlations in Study 1 (an abridged version of the table below focusing on anger is presented in the manuscript).

Supplementary Table 1. Means, standard deviations, and correlations in Study 1.

	M	*SD*	1.	2.	3.	4.	5.	6.	7.	8.	9.	.10	.11	.12
1. Relational mindfulness	5.91	0.69	1											
2. Relationship satisfaction	5.98	0.83	.26*	1										
3. Attachment anxiety	2.53	0.92	-.18*	-.13*	1									
4. Attachment avoidance	3.36	0.90	-.07*	-.05*	.13*	1								
5. Perceiver stress	1.88	1.21	-.15*	-.16*	.27*	.06*	1							
6. Partner stress	1.88	1.21	-.08*	-.08*	.10*	-.01	.33*	1						
7. Perceived partner stress	1.96	1.22	-.18*	-.19*	.16*	.03	.66*	.35*	1					
8. Perceiver anger	1.69	1.08	-.13*	-.17*	.19*	.01	.59*	.24*	.49*	1				
9. Partner anger	1.69	1.08	-.08*	-.08*	.07*	-.03	.24*	.59*	.26*	.24*	1			
10. Perceived partner anger	1.68	1.11	-.17*	-.16*	.12*	.02	.47*	.22*	.67*	.61*	.30*	1		
11. Perceiver happiness	4.43	1.39	.18*	.26*	-.11*	-.02	-.49*	-.24*	-.38*	-.47*	-.23*	-.35*	1	
12. Partner happiness	4.43	1.39	.09*	.16*	.03	.03	-.24*	-.50*	-.23*	-.23*	-.47*	-.21	.28*	1
13. Perceived partner happiness	4.31	1.36	.16*	.23*	-.08*	.01	-.44*	-.26*	-.43*	-.40*	-.27*	-.42*	.83*	.35*

Note. All variables were assessed on a scale of 1-7. Correlations represent zero-order correlations across all measurement time points. *p < .01

3. Stress and Happiness Accuracy and Bias Results in Study 1

In addition to anger, presented in the main manuscript, participants also rated their own and their partner's stress and happiness. We follow the same analytical approach as presented in the main manuscript in regards to perceptions of anger. Regarding stress, mindfulness was significantly negatively associated with directional bias (b = -.19, SE = .07, t = -2.89, p = .004) resulting in perceivers low in mindfulness significantly overestimated their partner's stress (b = .20, SE = .06, t = 3.23, p = .002), whereas perceivers high in mindfulness did not show directional bias (b = -.06, SE = .07, t = -.93, p = .355). See Supplementary Table 2.

Supplementary Table 2. Directional Bias, Tracking Accuracy, and Projection in the Perception of a Partner's Stress During Conflict Conversations.

Bias and accuracy of perception partner's emotions	*b*	*SE*	*t*	95% CI	*p*
			Stress		
Directional bias	.07	.05	1.610	[-.02, .16]	.111
Tracking accuracy	.10	.02	4.033	[.05, .14]	<.001
Projection	.45	.03	16.105	[.39, .50]	<.001
Effects of mindfulness					
Directional bias	-.20	.07	-2.887	[-.32, -.06]	.004
Tracking accuracy	-.05	.04	-1.299	[-.13, .03]	.197
Projection	.02	.04	.405	[-.06, .09]	.686

Mindfulness was not significantly associated with directional bias, tracking accuracy, nor projection of partner's happiness. See Supplementary Table 3.

Supplementary Table 3. Directional Bias, Tracking Accuracy, and Projection in the Perception of a Partner's Happiness During Conflict Conversations.

			Happiness		
Directional bias	-.12	.04	-3.191	[-.189 -.04]	.002
Tracking accuracy	.10	.02	5.331	[.06, .13]	<.001
Projection	.58	.02	26.206	[.53, .62]	<.001
Effects of mindfulness					
Directional bias	.09	.05	1.749	[-.01, .19]	.082
Tracking accuracy	.02	.03	.782	[-.043 .07]	.435
Projection	.001	.03	-.011	[-.06, .06]	.991

4. Models controlling for relationship satisfaction or attachment anxiety and avoidance in Study 1

For stress, the effect of mindfulness on bias remains significant when controlling for attachment (b = -.19, SE = .07, 95% CI [-.32, -.05], t = -2.715, p = .007) but marginally significant when controlling for relationship satisfaction (b = -.13, SE = .07, 95% CI [-.27, .001], t = -1.954, p = .052), and the effect of gender on the interaction between mindfulness and projection remained significant when controlling for relationship satisfaction (b = -.11, SE = .04, 95% CI [-.20, -.03], t = -2.581, p = .011) or attachment anxiety and avoidance (b = -.10, SE = .04, 95% CI [-.18, -.02], t = -2.358, p = .020). For anger, controlling for relationship satisfaction did not change the effect of mindfulness on bias (b = -.18, SE = .07, 95% CI [-.32, -.05], t = -2.709, p = .007) nor projection (b = -.10, SE =.05, 95% CI [-.19, -.02], t = -2.311, p = .022), and did not greatly change the effects of gender on the interaction between mindfulness and tracking accuracy (b = .11, SE =.04, 95% CI [.02, .19], t = 2.383, p = .019). Similarly, again for anger, controlling for attachment anxiety and avoidance did not change the effect of mindfulness on bias (b = -.22, SE = .07, 95% CI [-.36, -.09], t = -3.292, p < .001) nor projection (b = -.11, SE =.05, 95% CI [-.20, -.02], t = -2.407, p = .017), and did not greatly change the effects of gender on the interaction between mindfulness and tracking accuracy (b = .11, SE =.05, 95% CI [.01, .19], t = 2.236, p = .027).

STUDY 2

5. Models controlling for relationship satisfaction or attachment anxiety and avoidance

Including relationship satisfaction in the model did not change the effects of mindfulness on tracking accuracy (b = -.37, SE = 0.10, t = -3.638, 95% CI [-0.58, -0.17], $p < .001$), including in the model testing the effects by gender (i.e., the effect of mindfulness on tracking accuracy for men remain significant, b = -.50, SE = 0.17, t = -2.893, 95% CI [-0.85, -0.16], p = .004). Similarly, including both attachment anxiety and avoidance in the model did not change the effect of mindfulness on tracking accuracy (b = -.33, SE = 0.12, t = -2.838, 95% CI [-0.56, -0.10], p = .005), including in the model testing the effects by gender (i.e., the effect of mindfulness on tracking accuracy remain significant for men, b = -.63, SE = 0.19, t = -3.279, 95% CI [-1.02, -0.25], p = .0041).

6. Accuracy and bias across gender results

Supplementary Figure 1 displays the significant interaction with tracking accuracy plotting perceptions of partner's negative emotions centered on the partners' negative emotions across levels of partner's actual negative emotions (tracking accuracy) for those high (+1 SD) versus low (-1SD) in mindfulness. Similar to the effect of perceptions of anger for men in Study 1, tracking accuracy was significant only for people lower (-1 SD; *slope* = .49, *SE* = .07, $t = 7.706$, $p < .001$) but not higher in mindfulness (+1 SD; *slope* = .13, *SE* = .08, $t = 1.7358$, $p = .084$). Supplementary Figure 1 demonstrates the implications for understanding the links between mindfulness and directional bias. Differences across levels of mindfulness became greater the more partners were experiencing negative emotions revealing that men low (but not high) in mindfulness were increasingly likely to overestimate their partner's negative emotions when their partners were experiencing higher levels negative emotions. As found regarding perceived anger by men in Study 1, this pattern suggests that people low in mindfulness more accurately detected when their partners were experiencing higher levels of negative emotions but then tended to see their partners as experiencing more negative emotions than they were. More mindful people did not show this mix of tracking accuracy and bias suggesting they were less perceptually reactive to the presence of their partner's negative emotions.

Supplementary Figure 1. The effects of mindfulness on perceptions of partner's negative emotions across levels of partner's negative emotions in Study 2.

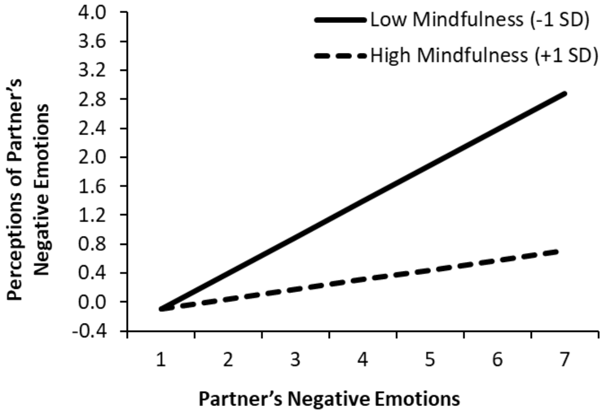

10. Results from supplemental analyses conducted using the FFMQ without the *observing* subscale

Supplementary analyses were conducted using the average of the FFMQ items minus the *observing* facet items. The pattern of results is similar to the original analysis with the full FFMQ scale. Specifically, mindfulness was significantly associated with directional bias and tracking accuracy as in the original analyses (see Supplemental Table 4 below). Decomposing these interactions revealed that perceivers low in mindfulness accurately tracked (-1 SD; *slope* = .51, *SE* = .07, $t = 7.669$, $p < .001$) but also overestimated their partner's negative emotions ($b = .21$, *SE* = .06, $t = 3.59$, $p < .001$), whereas perceivers high in mindfulness did not accurately track (+1 SD; *slope* = .12, *SE* = .07, $t = 1.626$, $p = .106$) or show bias in their perceptions of their partner's emotions ($b = -.01$, *SE* = .06, $t = 0.001$, $p = .999$). Again reflecting the patterns in the original analyses, differences across levels of mindfulness became greater the more partners were experiencing negative emotions, such that when partners were experiencing low levels of negative emotions (-1 SD) there was no difference in the perception of partner's emotions across levels of mindfulness ($b = 0.23$, *SE* = 0.14, $t = 1.728$, $p = .086$). However, when partners were experiencing high levels of negative emotions (+1 SD), a significant difference in the perception of partner's negative emotions across levels of mindfulness ($b = -.60$, *SE* = .14, $t = -4.567$, $p < .001$) revealed that perceivers low compared to high in mindfulness were more likely to overestimate their partner's negative emotions.

Supplemental Table 4. Directional Bias, Tracking Accuracy, and Projection in the Perception of a Partner's Negative Emotions (Study 2) During Conflict Conversations.

Bias and accuracy of perception partner's emotions	*b*	95% CI	*t*	*p*
**Study 2**				
Directional bias	.10	[.03, .18]	2.621	.010
Tracking accuracy	.31	[.21, .42]	6.154	< .001
Projection	.58	[.48, .68]	11.807	< .001
Effects of trait mindfulness (FFMQ without *observing* facet)				
Directional bias	-.20	[-.37, -.04]	-2.425	.016
Tracking accuracy	-.38	[-.56, -.19]	-3.998	< .001
Projection	-.01	[-.16, .16]	0.030	.976

Second, we also ran the model testing whether the effects differed across men and women (see Supplementary Table 5 below). Similarly to the original analyses, the effects of mindfulness on projection bias significantly differed across men and women ($b = .29$, $t = 2.815$, $p = .005$). As with the original analyses, mindfulness was significantly associated with projection bias for men ($p = .007$) but not for women ($p = .399$), and again showed that projection bias tended to be stronger for men high (+1 SD; *slope* = 1.01, *SE* = .15, $t = 6.657$, $p < .001$) compared to low (-1 SD; *slope* = .50, *SE* = .10, $t = 4.818$, $p < .001$) in mindfulness.

Also similar to the original analyses, there were gender differences in mindfulness on tracking accuracy across men and women, although the interaction effect was weaker ($b =$

-.20, $t = 1.925$, $p = .055$). Nonetheless, similar to Study 1, men low (-1 SD) in mindfulness showed significant tracking accuracy (*slope* = .68, *SE* = .10, $t = 6.604$, $p < .001$), whereas men high (+1 SD) in mindfulness did not (*slope* = -.08, *SE* = .11, $t = -0.682$, $p = .497$). Men's significant overestimation of their partner's negative emotions also was more pronounced for men low in mindfulness ($b = .40$, *SE* = .11, $t = 3.74$, $p < .001$) compared to men high in mindfulness (scores on average above zero; $b = .23$, *SE* = .11, $t = 2.18$, $p = .031$). Similarly to the original analyses, neither women low ($b = .87$, *SE* = .07, $t = 1.271$, $p = .206$) nor high ($b = -.10$, *SE* = .09, $t = -1.093$, $p = .276$) in mindfulness exhibited significant directional bias, and only women low (*slope* = .42, *SE* = .08, $t = 5.210$, $p < .001$) but not high (*slope* = .08, *SE* = .12, $t = 0.649$, $p = .518$) in mindfulness demonstrated significant tracking accuracy.

Supplemental Table 5. Accuracy and bias of negative emotions (Study 2) across men and women perceivers. The first column shows the coefficients testing whether gender moderated the effects, and the second and third columns illustrate the effects for men and women perceivers.

	Gender moderation				Men perceivers				Women perceivers			
Perception of partner's negative emotions	*b*	t	95% CI	*p*	*b*	t	95% CI	*p*	*b*	t	95% CI	*p*
**Study 2**												
Directional bias	.16	3.102	[.06, .26]	.002	.32	4.297	[.17, .46]	<.001	-.01	-0.114	[-.13, .11]	.909
Tracking accuracy	.03	0.576	[-.07, .12]	.565	.30	4.260	[.16, .44]	<.001	.25	3.437	[.11, .39]	<.001
Projection	.09	1.827	[-.01, .19]	.069	.76	8.469	[.58, .93]	<.001	.57	10.320	[.46, .68]	<.001
Effects of Mindfulness												
Directional bias	.01	0.102	[-.18, .20]	.919	-.16	-1.093	[-.46, .13]	.276	-.18	-1.699	[-.40, .03]	.092
Tracking accuracy	-.20	-1.925	[-.41, .01]	.055	-.74	-4.705	[-1.05, -.43]	<.001	-.33	-2.432	[-.60, -.06]	.016
Projection	.29	2.815	[.09, .50]	.005	.51	2.735	[.14, .87]	.007	-.08	-0.846	[-.27, .11]	.399

7. Relational Measures Study 1 and 2

Relationship Satisfaction (Rusbult et al., 1998)

1. I feel satisfied with our relationship.
2. My relationship is much better than others' relationships.
3. My relationship is close to ideal.
4. Our relationship makes me very happy.
5. (*Study 2 only*) Our relationship does a good job of fulfilling my needs for intimacy, companionship, etc.

Attachment anxiety and avoidance were measured with the Adult Attachment Questionnaire (AAQ; Simpson, 1990) by asking people to rate how they typically feel toward romantic partners in general.

Attachment Anxiety:

1. I rarely worry about being abandoned by others. *(reverse coded)*
2. Others often are reluctant to get as close as I would like.
3. I often worry that my partner(s) don't really love me.
4. I rarely worry about my partner(s) leaving me. *(reverse coded)*
5. I often want to merge completely with others, and this desire sometimes scares them way.
6. *(Study 2 only)* I'm confident my romantic partners would never hurt me by suddenly ending our relationship. (*reverse coded*)
7. *(Study 2 only)* I usually want more closeness and intimacy than my romantic partners do.
8. *(Study 2 only)* The thought of being left by my romantic partners rarely enters my mind. (*reverse coded)*
9. *(Study 2 only)* I'm confident that my romantic partners love me just as much as I love them. (*reverse coded)*

Attachment Avoidance:

10. I find it relatively easy to get close to others. *(reverse coded)*
11. I'm not very comfortable having to depend on other people.
12. I'm comfortable having others depend on me. *(reverse coded)*
13. I don't like people getting too close to me.
14. I'm somewhat uncomfortable being too close to others.
15. I find it difficult to trust others completely.
16. I'm nervous whenever anyone gets too close to me.
17. Others often want me to be more intimate than I feel comfortable being.

All items were rated on a 7-point scale (1 = *totally disagree* to 7 = *completely agree*)

11. Additional methodological information on laboratory conversation

The laboratory interaction studies were conducted following best common practices in relationships research (e.g., Overall et al., 2022; Visserman et al., 2022). Both studies were conducted in the psychology laboratories of the host institutions and were set up so that participants felt comfortable and the conversation would occur as naturally as possible. This included providing instructions prior to the conversation, asking whether either partner had questions, and ensuring privacy during the actual interaction and confidentiality of the data. Additionally, in Study 2, prior to discussing the chosen relationship issue, to help participants feel more comfortable having a discussion while being recorded, participants had a 5-minute warm-up discussion about how their week had been.

Participants were aware of the camera(s) and knew they were being recorded. In Study 1, one camera was set and the couple was seated to face away from it (but toward each other) to minimize distraction. In the laboratory used for Study 2, three cameras were mounted from the ceiling so they are not in the natural line of sight of participants' seated position.

In both studies, couples were told they would have 7 minutes to discuss and were briefed on how they would know the conversation time was done. In Study 1, the research assistant pressed record and then left the room. After 7 minutes, the research assistant knocked on the door and entered the room, marking the conclusion of the conversation. In Study 2, couples were asked to start discussing the topic via intercom. Seven minutes after the research assistants left the room, couples were informed over the speaker system that the recording was being stopped. The research assistant then knocked on the door and waited for the couples to indicate they were able to enter the room.

All topics (either a divergence of interest in Study 1 or an ongoing issue in Study 2) were chosen by participants prior to starting the conversation and participants briefly wrote them down before answering a short survey with some questions (e.g., importance of topic). In Study 1, each participant only listed one topic and couples decided together which topic to discuss during the conversation, while in Study 2, participants were asked to think of up to three issues they were currently facing in their relationship and were told that the researcher assistant would be choosing one person's relationship issue for them to discuss. Whose topic was chosen was counterbalanced across gender (e.g., couple 1 = woman's issue, couple 2 = man's issue). Issue selection criteria focused on identifying the most serious issue using the following information:

1. The issue that was ranked by the participant as number 1
2. The problem was rated as a 'serious problem' in the relationship (rated 5-7)
3. The problem was rated as not resolved (rated 1-3)
4. The problem with the greater discrepancy between 'Who needs to change the most in order to resolve this issue' **<u>IF</u>** both couples listed the same relationship issue and/or partner needs to change the most

In Study 1, each couple member separately rated how important the preference was for them (*M* = 5.16, *SD* = 1.0), how big the sacrifice would be (*M* = 4.48, *SD* = 1.34), how important they perceived the preference to be for their partner (*M* = 5.2, *SD* = 1.07), and how big they perceived the sacrifice to be for their partner (*M* = 4.60, *SD* = 1.36) (all measured on a scale from 1-7). In Study 2, for each issue listed, each couple member rated the severity (*M* = 4.95, *SD* = 1.42) and the extent to which the issue was resolved (*M* = 3.3, *SD* = 1.7) (both measured on a scale from 1-7). Participants completed these measures right after identifying the topic, but prior to starting the conversation

Below we provide the interaction protocol which was delivered by a trained research assistant during the laboratory session with each couple. For both studies, the interaction followed a baseline questionnaire completed separately by each couple member.

Study 1: Extra information regarding the video-taped conversation

"Now we would like you to individuate a situation of divergence of interests in your relationship. What we mean is that we would like you to individuate a situation in your relationship in which you and your partner have different preferences (e.g. on Saturday you would like to go to visit your family while s/he prefers to spend time with common friends; you would like to go on a trip to USA while your partner wants to go to Thailand; you would like to move to another country while your partner would like to stay in the Netherlands; you would like to have children while your partner would prefer not to; you would like to meet with a friend while your partner feels uncomfortable if you do so etc.). We are interested to see how couples discuss these situations and your interaction will be videotaped. Please discuss this issue as you would normally do at home. Again, I'd like to remind you that all of the data is confidential.You can talk about anything, but it needs to be a situation in which you have different preferences. It could be something current, or something you anticipate happening in the near future. It can be something you have already discussed or something you haven't discussed yet. We would like you to discuss this situation together for 7 minutes and try to come up with a solution (if after these seven minutes you do not reach a solution, that's also fine, just try to discuss the problem together and try to figure out what to do). Do each of you understand what kind of discussion topic we'd like you to select? If not, please ask the experiment to clarify."

Study 2: Extra information regarding the video-taped conversation

Script prior to warm-up conversation:

"The next part of the research will involve video-recorded discussions. These recording will be kept strictly confidential, and no identifying information will be kept with any of your data. The recording of your discussions will be securely stored on a password-protected hard drive in a locked filing cabinet, and only the researchers and trained coders, who have signed strict confidentiality forms, will view the discussions. The first discussion will be a 5 minute warm-up discussion about the events of your week. This is to just get you used to having a

conversation while being recorded and get you comfortable, so just have a chat about how your day or week has been. It is important that you don't discuss the relationship issue that has been selected for you to discuss next or any of the questionnaires you have filled out so far. I will just go out back and will let you know when I start recording through the intercom (i.e., "Please start discussing 'Events of the Week'"), at that point your 5 minutes starts. I will come on the intercom at the end again to let you know when I end the recording session (i.e., "I am going to stop recording now") – this does not mean you have to stop your conversation though. You can keep talking. I will just knock on the door, and you can let me in when you are ready."

Script prior to the conflict conversation (after warm-up):

"Now we will have a 7-minute discussion about the relationship issue that you answered questions about earlier. The aim of this discussion is to discuss the relationship issue and try to work together to come up with a solution or closer to a solution. We want you to just talk about the issue as you normally would. Perhaps think about the last time that this issue was a problem in your relationship and start from there. Please do remember that your discussion is completely confidential and only authorised people will view the discussion. I will again let you know when recording begins, which is when you can start your discussion and when I stop recording. Again, I will knock on the door and when you are ready you can let me in."
